# Editorial: Synthesis, Functionalization, and Clinical Translation of Pharmaceutical Biomaterials

**DOI:** 10.3389/fbioe.2021.707963

**Published:** 2021-06-21

**Authors:** Huimin Kong, Shixian Lv, Mingqiang Li, Jianxun Ding

**Affiliations:** ^1^Laboratory of Biomaterials and Translational Medicine, Center for Nanomedicine, The Third Affiliated Hospital, Sun Yat-sen University, Guangzhou, China; ^2^Department of Bioengineering, University of Washington, Seattle, WA, United States; ^3^Key Laboratory of Polymer Ecomaterials, Changchun Institute of Applied Chemistry, Chinese Academy of Sciences, Changchun, China

**Keywords:** biomaterial, pharmaceutics, bioengineering, biotechnology, clinical translation

In recent years, emerging biomaterials have made significant contributions to biomedical manipulation, pharmaceutical enhancement, and efficient and targeted delivery of agents for disease theranostics. In addition, the ongoing improvements in synthesis and functionalization of pharmaceutical biomaterials can bring innovations to practical clinical translation.

This invited Research Topic is provided through 38 articles, including 25 original research articles and 13 review articles, contributed by 245 researchers worldwide (Total views: 68,531 by June 7, 2021). These reviews cover Research Topics discussing both biomaterials and pharmacology. Some review articles provide guidelines for synthesis and functionalization of biomaterials, for example, proper material characteristics for nerve regeneration and integration (Guedan-Duran et al.) and biomaterial-mediated delivery strategies of small hydrophilic drugs (Li Q. et al.). The other reviews discussed various biomaterials applied to improve pharmacological efficacy and safety, such as implantable and injectable scaffolds for cancer immunotherapy (Li J. et al.), exosomes applied in visual and auditory systems (Jiang P. et al.), extracellular vesicle-based biomaterial scaffolds for bone regenerative medicine (Yan H. C. et al.), polymer-based scaffolds to repair spinal cord injury (Qu et al.), hydrogels with 3D printing to treat osteochondral and cartilage diseases (Dai et al.), supramolecular hydrogels for cartilage tissue engineering (Yan X. et al.), nerve guidance conduit biomaterials with additive manufacture for peripheral nerve regeneration (Song et al.), functional materials applied in treating chronic wound (Zhang et al.) or organ-on-a-chip technology (Ding et al.), nanocarriers to enhance drug bioavailability through transdermal delivery (Yu et al.) and nasal administration (Xu, Tao, et al.), and so forth.

In this Research Topic, a diverse of original works have given critical aspects of pharmaceutical biomaterials on their synthesis, functionalization, and potential clinical translation. Emerging pharmaceutical biomaterials are promising to establish diagnostic platforms or evaluate systems for diseases and lesions. For example, Wang et al. synthesized the ^18^F-labeled positron emission tomography molecular probe to predict vulnerable atherosclerotic plaques with the target to the α7-nicotinic acetylcholine receptor. Moreover, Jiang Q. et al. demonstrated a non-invasive detection of hepatocarcinoma tumor margin by the nanobubble-mediated ultrasound imaging. Fluorescence imaging is another promising diagnostic toolkit. An emission-enhanced near-infrared fluorescent nanoparticle was presented, containing matrices with polystyrene moiety and AIEgen and showing application potentials for *in vivo* three-photon brain imaging (Du et al.). Moreover, artificial models for biomimetics offer opportunities to understand and evaluate biomedical effects. For example, the tunable-structured composite hydrogels with gelatin-methacrylate (GelMA) and hydrolyzed collagen were generated with three-dimensional bioprinting and further applied in a tumor-on-a-chip system to model tumor micro-environment and evaluate cancer invasiveness (Chen et al.). Besides, growing polymer vesicles with Belousov-Zhabotinsky reaction-coupled polymerization-induced self-assembly were illustrated as the biomimetic chemical oscillation promising for cell model developments (Lu and Guo).

Apart from diagnostic applications, advanced biomaterials with outstanding therapeutic outcomes are also included. Among them, some biomaterials can manipulate cell behaviors, for example, an anisotropic inverse opal for neural regeneration *via* regulating the growth of neural stem cells (Xia et al.), a prepared tin dioxide nanoparticle with high cytotoxicity in oral cancer cells *via* resisting cancer activities (Li H. et al.), an injectable gelatin hydrogel for intracerebral hemorrhage recovery *via* suppressing the inflammation (Xu, Duan, et al.), a biphasic double-network hydrogel majorly containing glycol chitosan, sodium alginate, and bioactive glass for osteochondral repair (Liu B. et al.), etc. Significantly, nanoparticle-based drug delivery systems are manipulated to optimize their therapeutic efficacies. Nanocarriers have been prepared and functionalized to achieve high/stable encapsulation, efficient and targeted delivery, controlled release of cargos. Typically, proper-selected excipients that drive excellent stability for polymer microparticles were reported for further clinical translation to antigen-specific immunotherapy (Gosselin et al.). Furthermore, a stable astragalus polysaccharide nanoparticle improved the activity and bioavailability of the single drug to inhibit cerebral thrombosis (Sun Q. et al.). Additionally, PEGylation of deferoxamine, a drug to treat iron-overload-related diseases, was verified to be more stable and lower cytotoxic in a stroke mouse model (Xu, Sun, et al.).

To perform optimized effects in dysfunctional sites, efficient, and targeted delivery is needed to guarantee therapeutic specificity. For instance, an implantable porous poly(D,L-lactide-*co*-glycolide) microsphere (PLGA MP) loaded with mesenchymal stem cells was developed for modular tissue engineering (Simitzi et al.). This cellularized PLGA MP achieved efficient delivery of MSCs and significant osteogenic or chondrogenic differentiation. Likewise, hydrogels complexed with a soft polymer matrix and well-carried bone morphogenetic protein receptor inhibitors were proved to promote neural differentiation of MSCs (Sun, Xu, et al.). While for targeted delivery, a copolymer-mixed micelle with hyaluronic acid and Pluronic F68 to co-deliver docetaxel and programmed cell death ligand-1 antibody, which showed excellent efficacy of tumor-targeting and immune-chemotherapy (Zhou et al.).

Proper drug release profiles are essential for the drug delivery system toward different therapeutic goals. For stimuli-responsive drug release, a reactive oxygen species (ROS)-sensitive micelle was developed for the co-delivery of dexamethasone and cartilage-derive morphogenetic protein 1 to treat osteoarthritis (Wu et al.). Likewise, a magnetism-responsive and celecoxib-loaded gelatin hydrogel was applied to deliver celecoxib to achieve synergistic pulsed electromagnetic field and drug therapy of tendon tissue injury (Wang J. et al.). Moreover, a synthesized thermo-sensitive hydrogel containing oxaliplatin and alendronate effectively enhanced *in situ* osteosarcoma therapy (Sun, Li, et al.). An antiviral supramolecular hydrogel was constructed for sustained release *via* charge-mediated co-assembly of amphiphilic peptide and polymyxin B antibiotics (Xu L. et al.). This supramolecular hydrogel realized sustained release of antibiotics and effective bacteria inhibition.

Furthermore, biomaterials have been explored to synergize with other therapy strategies. As an example, the combination of selenium nanoparticle and radiotherapy significantly inhibited the tumor growth of lung cancer, providing a new anti-cancer method (Tian et al.). Besides, combined biomaterials have been developed to perform double therapeutic effects. An injectable GelMA hydrogel and magnesium-zinc alloy complex for accelerated bone regeneration to repair the calvarial defect (Wang W. H. et al.).

Lastly, the choice of different drugs according to drug susceptibility, drug resistance, and compatibility of medicines is essential in clinical applications. For instance, the strain distribution changes and drug susceptibility of invasive fungal strains, providing references for selecting sensitive antifungal drugs in patients with systemic internal diseases (Zeng et al.). Besides, the combination of hydroxyasiaticoside and praziquantel performed a significant therapeutic effect *in vivo* to treat schistosomiasis-induced hepatic fibrosis (Fang et al.). Furthermore, the ongoing drug discovery offers ideal candidates to be bioengineered for future clinical treatments. For example, cepharanthine, a natural alkaloid extracted from the genus Cephalophyllum, showed efficient cathepsin B targeting therapy for cutaneous melanoma (Liu Q. et al.). Additionally, Rhizopus nigrum polysaccharide EPS_1−1_ was studied as a potential target of drug or therapeutic adjuvant for hepatocellular carcinoma (Chu et al.).

Overall, the current Research Topic reports the fundamental research and clinical applications of various pharmaceutical biomaterials. This interdisciplinary hot issue showed the inexhaustible power to tightly connect biomaterial innovation with clinical translation, greatly benefiting biomaterials, bioengineering, biotechnology, and pharmaceutics.

## Author Contributions

All authors listed have made a substantial, direct and intellectual contribution to the work, and approved it for publication.

## Conflict of Interest

The authors declare that the research was conducted in the absence of any commercial or financial relationships that could be construed as a potential conflict of interest.

